# Reducing fragmentation of primary healthcare financing for more equitable, people-centred primary healthcare

**DOI:** 10.1136/bmjgh-2024-015088

**Published:** 2025-01-14

**Authors:** Agnes Gatome-Munyua, Susan Sparkes, Gemini Mtei, Martin Sabignoso, Prastuti Soewondo, Pierre Yameogo, Kara Hanson, Cheryl Cashin

**Affiliations:** 1 Results for Development Institute, Nairobi, Kenya; 2 World Health Organization, Geneva, Switzerland; 3 Abt Associates, Dar es Salaam, Tanzania, United Republic of; 4 Independent Consultant, Buenos Aires, Argentina; 5 Ministry of Health, Jakarta, Indonesia; 6 Ministry of Health and Public Hygiene, Ouagadougou, Burkina Faso; 7 Department of Global Health Development, London School of Hygiene and Tropical Medicine, London, UK; 8 Results for Development Institute, Washington, District of Columbia, USA

**Keywords:** Health economics, Health policy, Health services research, Health systems

## Abstract

Despite primary healthcare (PHC) being recognised in global declarations—Alma Ata in 1978 and Astana in 2018—and prioritised in national health strategies, chronic under-resourcing of PHC persists in most low-income and middle-income countries. More public spending is needed for PHC, but macrofiscal and political constraints often limit the ability of governments to allocate more public resources to PHC. Under-resourcing has been compounded by fragmented and rigid funding flows, which are inefficient and may erode equity, quality of care and public trust in PHC.

This article explores the drivers of fragmentation in PHC financing—low public spending, which results in over-reliance on external sources to fund critical health interventions, and the proliferation of new financing schemes that do not take a system-wide view or adhere to the principles of universality. It then highlights some of the possible consequences of this fragmentation for the efficiency, equity and effectiveness of service delivery.

Four countries—Argentina, Burkina Faso, Indonesia and Tanzania—are used to illustrate practical steps that may be taken to minimise the consequences of fragmentation in PHC financing: (1) consolidating multiple coverage schemes, (2) avoiding further fragmentation, (3) harmonising health purchasing functions and (4) streamlining funding flows to the provider level.

The country examples reveal lessons for policy-makers grappling with the consequences of fragmented PHC financing. The paper concludes with a research agenda to generate additional evidence on what works to address fragmentation.

**Summary box**

- The authors explore the drivers of primary healthcare (PHC) financing fragmentation and how fragmentation impacts the flow of resources, with consequences for the efficiency, equity and effectiveness of PHC.
- The article presents approaches used to minimise the negative consequences of PHC financing fragmentation from Argentina, Burkina Faso, Indonesia and Tanzania and reveals four lessons: (1) the importance of avoiding new schemes or funding flows that bypass government systems; (2) the need to actively manage the political economy that worsens and entrenches fragmentation; (3) the opportunity to use strategic purchasing as an entry point to defragment funding flows to PHC and (4) how defragmenting a small part of the system can be an important catalyst to drive more efficient and effective PHC financing.
- There is scope for further refinement of the concept of fragmentation of financing, for PHC and more generally, including consideration of when fragmentation is inherently negative, the consequences of fragmentation and how these can be addressed through policy design.
- This paper is primarily targeted at those influencing health financing arrangements in low-income and middle-income countries; policy-makers in countries initiating reforms to improve how they purchase PHC; researchers who may contribute further evidence on how to address fragmentation and its potentially negative consequences. It seeks to raise awareness of development partners and policy-makers on the harm created by new programmes that do not consider a system-wide lens or conform to the principle of universality.

## Introduction

 There is consensus that strengthening primary healthcare (PHC) is a cornerstone to achieving universal health coverage (UHC) and improving population health.^1^,^7^ Despite PHC being recognised in global declarations—Alma Ata in 1978 and Astana in 2018—and incorporated in national health strategies, this commitment has not been fully realised. PHC can be characterised as a whole-of-society approach to organising national health systems to bring services closer to communities. This paper uses a narrower definition, building on the work of the Lancet Global Health Commission for Financing PHC, classifying PHC as the service delivery platforms from which a broad range of essential and cost-effective interventions are provided at the community level.^8^ The service delivery platforms are defined by each country, usually including individual and networked primary care facilities and hospitals, and in some cases, community health services.

The Lancet Global Health Commission on Financing Primary Health Care recently highlighted that PHC is not funded adequately or effectively by low-income and middle-income country governments to provide equitable access to PHC.^8^ Domestic public sources are essential for moving towards UHC, but most PHC funding continues to be sourced from donors and out-of-pocket payments in low-income and middle-income countries.^8 9^ PHC providers are often under-resourced and face shortfalls in human resources, equipment, commodities and medicines, all of which erode the quality of care and public trust in PHC.^10^ The under-resourcing of PHC is compounded by fragmented and rigid funding flows and operational arrangements. Critical health interventions such as prevention and treatment of HIV/AIDS, tuberculosis, malaria and immunisation are often reliant on external sources of funding, frequently allocated vertically to specific programmes. This can cause misalignments, duplication and overlaps in components of the health system,^11^,^13^ working against equitable, integrated PHC service delivery platforms and efficient use of limited resources.^14^,^17^ Fragmented PHC financing poses a significant barrier to operationalising and prioritising people-centred PHC.^8^ Nevertheless, while the problem of fragmentation in health financing is well recognised, it remains under-researched and poorly defined.^18^,^21^

In this article, we apply the Bossert *et al* definition of health system fragmentation as the ‘division without explicit means of coordination’ to health financing functions (eg, revenue sources, pooling, eligibility, benefits, premiums and payments) or agents (eg, purchasers and providers) in a health system or subsystem.^22^ This is similar to the definition of health financing fragmentation proposed by McIntyre *et al*, as the existence of a large number of separate funding mechanisms (eg, many small insurance schemes) and a wide range of healthcare providers paid from different funding pools.^23^ Fragmentation creates barriers to redistribution of pooled funds and can contribute to inefficiency in the health system due to duplication in the number of agencies required to manage pools and purchasing^24^ and hamper the equitable distribution of resources from a given level of resources.^25 26^ Importantly, addressing potential fragmentation does not only have to come through changes to the health financing system. Some countries such as Japan, the Netherlands and South Korea have used regulation and institutional arrangements to delineate and coordinate functions of different financing arrangements.

The central premise of this paper is that more careful design of purchasing functions may dampen, and in some cases address, the negative consequences of fragmentation at the system level. While it is common to link fragmentation to the pooling function of health financing systems, we argue here that the purchasing function determines the interface between fragmented PHC financing and service delivery. Purchasing is the transfer of pooled funds to providers for delivery of health services and can be considered strategic if it links evidence and information about population health needs to health provider performance and payment methods. Purchasing may be one way to address the fragmentation of PHC financing by designing benefit packages to include entitlements for PHC and linking entitlements to payment via output-based provider payment.

This paper characterises the main drivers of fragmentation of PHC financing in low-income and middle-income countries and highlights some of the possible consequences of this fragmentation for the efficiency, equity and effectiveness of service delivery. It shares the experiences of several countries that are addressing fragmentation through approaches that facilitate the flow of resources to providers and organise the purchasing function of health financing systems in a more coordinated, efficient and equitable manner. Finally, a research and learning agenda is proposed to further refine the concept of fragmentation, understand where and when fragmentation can be useful or detrimental for health systems and provide more evidence for countries to mitigate the negative consequences of fragmentation in the short term while the fundamental root causes of fragmentation are addressed over the longer term.

## Drivers of PHC financing fragmentation

Low public spending on health has led to piecemeal efforts to expand coverage through reliance on external funds and the introduction of new schemes. These new schemes, along with donor funding flows, often have their own rules and allocation criteria that can exacerbate fragmentation and work against the principles of universality and system-wide approaches. Universality carries with it a strong equity dimension and a mandate to ensure access for the poor and most vulnerable in order to improve their health and welfare.^27^,^29^ System-wide approaches aim to progress towards the UHC objectives by engaging the entire health system.^25 30^ The system-wide approach requires programmes or initiatives that address a subset of health conditions, or only some segments of the population, to consider coherence and mitigate distortions with other financing flows, including payment methods, pooling dynamics and overall governance structures. New schemes that are designed with the intention of reducing coverage gaps, but which do not take a system-wide view, or adhere to the principle of universalism, have the potential to exacerbate potential negative consequences of fragmentation.

### Low public spending

The driver of much of the fragmentation in PHC financing can be traced to low public spending on health overall, along with the low share of that public spending reaching PHC facilities. Government spending on PHC is about US$3 per capita on average in low-income countries and US$16 in lower-middle-income countries, while private spending in the form of out-of-pocket payments by households is two to three times higher than government spending.^8^ High out-of-pocket spending puts a disproportionate burden on vulnerable households and reduces the likelihood of seeking care when needed.^31^ Low public spending also reduces the share of funding that can be allocated strategically by public purchasers and has led to over-reliance on external sources to fund critical health interventions, as well as the proliferation of new insurance schemes that benefit the privileged minority, and do not address coverage gaps.^32^

### Reliance on donor funding for PHC services

External funding for PHC is often channelled through dedicated health programmes that ring-fence the funds for specific preventive or disease-specific services, which can add to fragmentation across all levels of the health system.^33^ These programmes were often introduced to support the prioritisation and delivery of critical health interventions but in doing so bypass the mainstream system with separate or parallel structures for service delivery, supply chain, funding flows, information systems, health workers and accountability mechanisms.^34^ In some cases, this ‘vertical’ approach has contributed to improved child and maternal mortality, reducing morbidity and mortality due to preventable diseases such as polio, childhood pneumonia, meningitis, malaria, tuberculosis and HIV/AIDS.^35^,^37^ However, these vertical health programmes have had unintended consequences across the system, with multiple donor-funded projects that have separate, often overlapping and duplicative, resources for each disease or intervention area.^38^,^40^ As an example of the potential scale of donor-driven fragmentation in a single country, a recent public expenditure review in Tanzania found that in 2017 alone, there were 504 separate health projects funded by development partners.^41^ As another example, in 2020 in Malawi, 55% of total health expenditure was funded externally across 166 financing sources and 265 implementing partners; contributing to poor coordination and misalignment between government priorities and donor projects.^42^ The proliferation of vertical health programmes, supported by separate funding flows, can divert resources towards individual services or populations via parallel/vertical service platforms, at the expense of integrated PHC service delivery platforms.^10^,^17^ This dynamic can weaken the foundations of health systems and prevent PHC services from meeting all the health needs of populations.

### New financing and coverage schemes

Many low-income and lower-middle-income countries have introduced new coverage schemes^43^ to tap into dedicated funding sources, both domestic and external, to close the gaps in public spending and address underprioritised conditions and populations, or to introduce more flexibility into funding flows. We define ‘coverage schemes’ as a pooled source of funds that gives access to a defined set of services to a population with defined eligibility with specific mechanisms for purchasing those services from health providers. These new health financing schemes may include community-based or social health insurance, performance-based financing (PBF) schemes, voucher systems, etc. Each of these has different factors that led to their introduction. For example, community-based health insurance schemes were introduced to increase access to services for informal sector workers that could be aggregated in similar groups, for example, farmers or communities living in the same location, while social health insurance was introduced for government workers and/or formal sector workers and ties coverage entitlement to the ability to make contributions from employees and employers. PBF and voucher schemes were introduced to address health system challenges including low coverage of critical health interventions, poor quality of health services, unmotivated health workers and weak accountability arrangements. Some of these schemes have resulted in improvements in access to health services, for some segments of the population targeted by these schemes.^44^,^47^ The impetus for these new schemes can be from external donors, government-funded programmes, changes in government policy or presidential declarations.^48^,^50^

Setting up these new schemes may have been deemed necessary to reach certain populations, fund necessary public health interventions and to accelerate progress on achievement of objectives, including those related to the Millenium Development Goals. They mostly have similar objectives to reach specific populations with specific priority services or to protect some populations from user fees. But by virtue of targeting specific populations or health conditions, many brought fragmentation in eligibility requirements, entitlements and benefits packages, contracting and payment mechanisms for providers, and their own reporting and monitoring systems. This is worsened when there are separate budget execution rules, including provider payment systems and reporting processes for these funds. The funds designated for PHC are often tied to specific services and populations, arrive at different bank accounts and the funds may not even reach PHC providers and are subject to different spending and reporting requirements. The inefficiency created by this fragmentation may have consequences for equitable and affordable access to quality health services for all.^6^ These schemes, like many externally financed programmes, often circumvent the government budget process and the public financial management (PFM) system. In doing so, they may contribute to inequitable resource distribution. In many countries, coverage gaps and overlaps coexist, resulting in a patchwork of partial coverage, mixed sets of rules and incentives for providers, and politically entrenched interests that are difficult to undo.^6 51^

## What are the consequences of this fragmentation to providers and populations they serve?

The effects of fragmentation on health providers have not been well investigated and so here we provide a set of hypotheses on the consequences of fragmentation for providers based on authors’ experience and the literature on health system inefficiencies arising from multiple and parallel pools and service delivery platforms.

When health financing systems are fragmented, equity in resource allocation may suffer, which may impact the most vulnerable. Inefficiencies may also abound due to the high costs of duplicative and parallel systems (eg, information systems, supply chain, trainings, supervision and data systems).^52^ PBF and vertical programmes have been important channels to achieve improvements in health indicators^4553^,^56^ and sometimes these PBF and vertical programmes have evolved and been integrated with government systems to strengthen purchasing.^39 57^ But where this integration has not happened, inefficiencies persist.^2657^,^59^

This fragmentation has resulted in weak purchasing. The multiple uncoordinated purchasers reduce the purchasing power that can be harnessed from fewer purchasing agencies to improve resource allocation and create financial incentives to providers for high-quality health services. Instead, each purchaser has its own rules for defining the benefit packages, contracting providers, and provider payment and any improvements to these processes may not translate to system-wide improvements because they only affect a small section of the health system.

**Figure 1:**
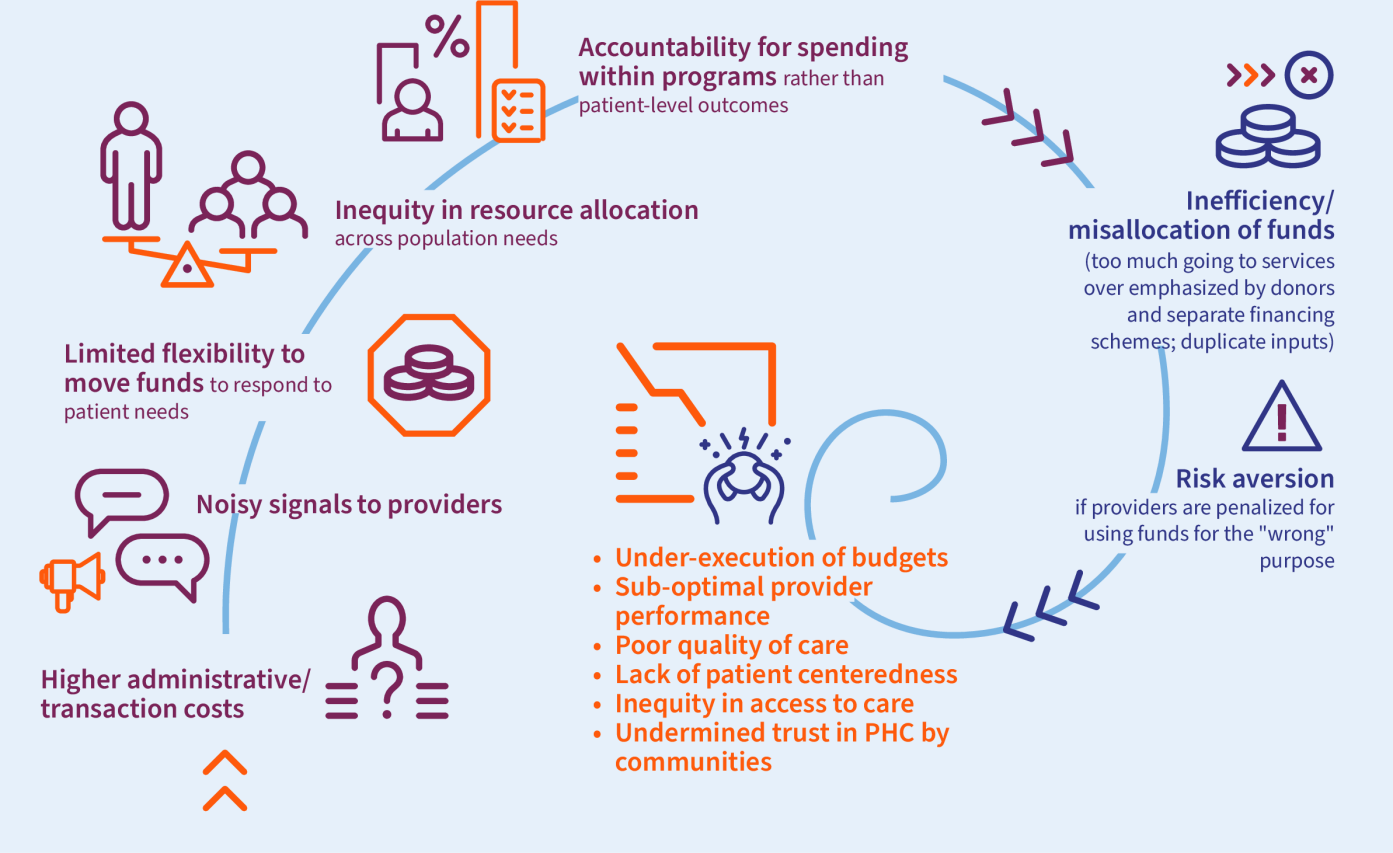
Proposed effects of fragmentation to health providers and the communities they serve. PHC, primary healthcare.

At the provider level, fragmentation may lead to a downwards spiral (figure 1) where providers receive noisy and contradictory signals from the separate funding channels with different services, population groups, rules for using resources and accountability mechanisms. Because of the different rules related to different funding streams, providers may be unable to prioritise and reallocate resources based on the changing needs of the populations they serve. This rigidity may be even more severe when funding is based on predefined inputs or externally determined priorities, and less on the population size and health needs. For those allocations to PHC based on fixed inputs in the form of line-item budgets or in-kind contributions, providers may have limited flexibility to use these funds and face strict accountability measures with sanctions for non-adherence.^21^

This may result in providers being unable to make holistic decisions about how to spend limited funds on the priorities of the populations they serve. Constraints in how providers can use funding combined with disbursement delays may lead to under-executed budgets.^51 60^ This means that already limited health budgets may go unspent, which may trigger a perverse cycle where budgets are reduced further to health providers that are already underfunded. Fragmentation may also create a heavy administrative burden for providers due to uncoordinated and duplicative inputs and reporting functions. This may further erode efficiency and the quality of health service delivery. All these factors may be compounded by other health system failures such as human resource shortages and supply chain failures, among others. This may all contribute to undermining trust in PHC services among the population, which may further reduce utilisation and increase bypassing of PHC to seek care at higher levels of the system, pulling even more funds away from the PHC level.

## Practical steps countries are taking to address fragmentation of PHC financing

Given the response to low levels of public spending that drive fragmentation in PHC financing, the Lancet Global Health Commission on PHC Financing made recommendations to fundamentally strengthen and prioritise public funding for PHC through progressive universalism with PHC at the centre of integrated service delivery.^8^ In the ideal setting, countries aim for a single pool or small number of pools with well-aligned purchasers with clearly articulated roles defined in a governance framework. This vision remains a long-term aspiration in many low-income and middle-income countries given the macrofiscal and political economy constraints they face. Countries have the option of refusing new donor-driven financing schemes that increase fragmentation. However, the very real fiscal and political constraints facing many low-income and middle-income countries mean that it is difficult to turn away new funds.

Many countries are taking short-term steps to reduce the negative consequences of fragmentation while addressing the root causes remains a longer-term goal. Some countries, such as Indonesia, Rwanda and Thailand, have been able to consolidate multiple pools or better align multiple purchasers with clearly articulated roles defined in a governance framework.^61 62^ However, there are contextual and political factors that can prevent full integration or harmonisation of purchasing arrangements and streamlining funds flow to the PHC provider level.

Among donors, sector-wide approaches, the Paris Declaration on Aid Effectiveness and other coordination initiatives have aimed to improve aid effectiveness through greater alignment and coordination with government priorities. These top-down, donor-driven mechanisms have not borne fruit, however, in terms of defragmenting overall health financing systems across countries.^14 63^ The Lusaka Agenda, launched in December 2023, represents the most recent effort to push for reform to the global health architecture to align and support more sustainable approaches to strengthening PHC.^64^

Yet there are several practical entry points to reduce the effects of fragmentation, some of which may not require significant additional resources or major organisational change. For example, countries can take a stepwise approach to harmonise purchasing functions (such as benefits packages, provider payment and performance monitoring) to clarify and align signals to providers, improve equity and streamline funding flows at the provider level. To illustrate these practical options, we share the experiences of four countries and the steps they are taking to address the consequences of fragmentation by consolidating multiple coverage schemes (Indonesia), avoiding further fragmentation (Burkina Faso), harmonising purchasing functions (Argentina) and streamlining funding flows to the provider level (Tanzania).

Importantly, the design of a scheme may contribute to or detract from UHC objectives for the broader population.^25^ The country examples presented here are characterised by their choice to expand coverage by designing policy interventions with the principle of universality and a system-wide lens. In these four countries, intervention design progressively supported improved equity in access to good quality health services and/or financial protection for the entire population and not limiting these to the few initial beneficiaries of the scheme. The design may have started with guaranteeing access to a subset of the population, as in Argentina and Burkina Faso, or coverage for an explicit set of services as in Tanzania, but overall, the design was meant to generate improvements for the population over time. All the countries take a system-wide view of the reform, illustrated in Argentina and Tanzania which use the PFM system to improve the flow of resources. By consolidating schemes in Indonesia, the focus on improvements in access was broadened to all the beneficiaries of the various insurance schemes rather than focusing on beneficiaries of a single scheme. The sections below describe these policy interventions and the unfinished agenda to continue improvements and advance UHC.

### Indonesia: consolidating multiple schemes and unifying purchasing functions

In 2014, Indonesia consolidated multiple public insurance schemes (civil servants, formal sector workers’ health benefits and health assistance schemes for the poor) under a single pool through the national insurance programme Jaminan Kesehatan Nasional (JKN) managed by a single insurer Badan Penyelenggara Jaminan Sosial Kesehatan (BPJS-K). Before 2014, the insurance schemes were managed independently with different benefits and provider payment systems. The move to JKN reduced fragmentation in the funding pools and harmonised the main purchasing functions, meaning that JKN funds flow to providers for services in one unified benefits package, through a single provider payment system and set of performance monitoring requirements, for more than 80% of Indonesians covered by JKN. Alongside JKN funds, other resource flows continue to support PHC: (1) From Ministry of Health (MOH) to provinces and district health officers for their delegated health functions and health programmes and directly to Pusat Kesehatan Masyarakat or Community Health Centers (Puskesmas); (2) central government transfers directly to provincial and district governments which may allocate local budgets to health and (3) funds from donors for prioritised programmes—HIV/AIDS, tuberculosis, malaria and immunisation programmes.^65^

Indonesia has over 10400 Puskesmas spread throughout the archipelago. Puskesmas receive funds from multiple sources to support their operations, including capitation payments from JKN and MOH budget funding.^66^ Despite the consolidation in public insurance pools brought about by JKN, funding still flows to PHC providers through multiple (up to five) streams to fund different sets of PHC services or inputs and with different financial management rules, which continues to undermine the coherence of the health financing system.^65^ Puskesmas receive financial and in-kind support from other channels such as local government funding to cover their operational costs, staff salaries, and other essential expenses and out-of-pocket payments from patients. MOH budget funding is designated for specific public health initiatives such as disease prevention (screening, immunisation, etc), health promotion and other public health programmes. Puskesmas have the autonomy to use funds from BPJS-K, user fees and budget support from MOH and local government, within the guidance provided, but in practice, these funding sources are managed separately at the facility level because they serve different purposes and have distinct accountability requirements, creating an administrative burden on providers.

Despite the fragmented sources of funding, some budget consolidation has been implemented to overcome potential barriers to using these funds efficiently at the PHC level. Puskesmas have some discretion to allocate the available resources to address health problems at the community level. For example, public funds are used for tuberculosis screening activities to avoid JKN duplicating tuberculosis screening in the benefit package, and patients who are diagnosed can receive immediate follow-up curative care funded through JKN. This allows Puskesmas to have a certain level of financial independence and decision-making power in using their resources creatively to respond to needs within the constraints of what each purchaser covers.

### Burkina Faso: avoiding further fragmentation and harmonising purchasing functions

Burkina Faso has a long history of user fee exemption with a policy to reduce direct costs of obstetric and neonatal care passed by Parliament in 2006 and pilots thereafter from 2008.^67^ Analysis of the initial user fee removal pilots revealed implementation gaps including continued charges to women despite the elimination of user fees.^67^ The policy did not eliminate financial barriers to accessing obstetric care.^67^ To address these challenges, Burkina Faso introduced Gratuité in 2016—a user fee replacement policy and a mechanism of prepayment of public funds to PHC providers for a defined package of services targeted at women and children under 5 years of age offered at PHC level. Gratuité has been credited with improving coverage of facility-based deliveries, increased consultations for children under 5 years and reduced out-of-pocket payments.^68 69^ In 2019, the package was expanded to include family planning services.

In 2018, the World Bank proposed extending its funding for a PBF scheme which had been ongoing since 2011.^70^ The PBF programme had implementation challenges and was perceived as expensive by country stakeholders.^71^ For example, a study by Beaugé *et al* found that targeting the ultra-poor with the PBF programme to access free healthcare services did not result in increased access to services for this group.^72^ Burkina Faso’s MOH preferred to avoid extending PBF and negotiated to redirect donor resources toward improving the existing Gratuité scheme through a restructuring of the World Bank project.^73^ In this case, it was more appropriate to pool donor resources and expand coverage to more women and children in underserved geographical areas through the government’s Gratuité system, than to continue with PBF that would perpetuate fragmentation of PHC financing. Since May 2023, World Bank funds are channelled into coverage of PHC in the Gratuité benefit package in eight regions (Boucle du Mouhoun, Sahel, North, Centre-North, Centre-West, South-West, East and Centre -East) and the government budget covers five other regions of the country (Centre, Hauts-bassins, Cascades, Centre-Sud and Central Plateau).

In addition, the MOH and World Bank harmonised some purchasing functions, documented through a procedure manual for Gratuité. This includes a common governance structure through a Task Force for strategic purchasing and steering and monitoring committee for Gratuité and other health services to avoid fragmentation. There is a single purchaser—the technical secretariat of health financing reforms—within the central department of the MOH, reporting directly to the Minister’s office. Both sources of funds—World Bank and the government budget—use the same billing rates, claims and payment system, and mechanisms for verification and monitoring of the implementation of Gratuité, validating the monitoring reports. The unfinished agenda in improving the purchasing functions is demonstrated by efforts to introduce contracting in the government budget-financed regions and digitalising the claims process in the World Bank-funded regions.

### Argentina: harmonising purchasing functions

In 2004, the Argentina MOH launched Plan Nacer (renamed Programa Sumar in 2012) to reinforce the public health system managed by the provinces and municipalities. At the time, Argentina was facing an economic crisis and contraction of the workforce. Many Argentinians were pushed under the poverty line and lost their social health insurance coverage. The objective of Plan Nacer was to address the coverage gaps, starting with the northern provinces which had the worst indicators. The federal MOH implemented conditional budget transfers with financial assistance from the World Bank. Although the source of funds was new, and a separate project management unit was created, the funds were channelled through the government’s financial systems. Conditional transfers have been faulted as being restrictive and a tool for central government control, but when well designed, with clear performance metrics, they are useful to align the subnational governments to national policy objectives.^74 75^ The budget transfers constituted less than 1% of the average annual provincial health budgets—and linked transfers to results to strengthen the strategic purchasing function in all provinces and improve the coverage of prioritised PHC services, with the goal of reducing neonatal morbidity and mortality.^76^ Since 2009, these transfers have been cofinanced by the provinces. The package of services is harmonised across the country and has grown over time to gradually include new services and cover new population groups. The programme was expanded in 2020 with the addition of beneficiaries above 64 years of age.^77 78^ At present, it covers the whole population without formal health insurance, estimated at approximately 21 million beneficiaries.

Transfers to provinces and municipalities are made via capitation payments based on enrolment of the eligible population who received a preventive service in the last 12 months and provinces’ performance (achievement of health outputs and outcome indicators). Provinces also receive an additional fixed amount based on their jurisdiction’s life expectancy (equity criteria). Transferred funds can only be used by provincial ministries of health to purchase health services for an explicit package from public providers for the enrolled population. Provider autonomy to allocate and use funds was expanded to increase health workers’ motivation and improve the volume and quality of PHC. Strategic purchasing was strengthened by defining a specific benefit package of priority services and linking resources to the delivery of these services. Evidence was used to track the achievement of the programme and fine-tune over time, increasing the scope of coverage to include more population groups. Using output-based payment—capitation—as a basis for the conditional transfers also resulted in more equitable resource allocation across regions based on the population rather than the previous system of input-based budgeting. Although only a small share of funding is channelled through Programa Sumar, impact evaluations have demonstrated significant improvements in service utilisation and health status for women and children under 5 years and demonstrated that the programme is highly cost-effective.^79^

### Tanzania: streamlining funds flow to the provider level

Tanzania’s on-budget donor resources are pooled into the Health Sector Basket Fund (HSBF). Since 2018, Tanzania has used an equity-based formula to allocate the HSBF through a unified flow of funds to front-line PHC providers through direct health facility financing (DHFF). Before DHFF, PHC facilities were not recognised in the Chart of Accounts (COA) as accounting units and therefore could not receive HSBF funds directly. HSBF and health insurance reimbursements from the National Health Insurance Fund and the improved Community Health Funds were disbursed to the local government authorities (LGAs) which had budgetary oversight and responsibility over PHC facilities. While LGAs received about a half of public health resources, about half was allocated to PHC facilities but 80% was spent on salaries and allowances leaving very little for other priorities.^80^ The DHFF reform incorporated the PHC facilities in the COA to receive HSBF funds directly through the government’s PFM systems. The objective of this reform was to overcome bottlenecks to resources flowing to PHC facilities, increase access to high-quality maternal and child health services, and improve accountability and responsiveness of PHC to increase health seeking at PHC facilities.^81^

By pooling on-budget contributions and sending the funds directly to providers, through the existing PFM system, DHFF has increased the flow of resources to PHC facilities. HSBF resources are allocated via a population-based capitation formula that allocates resources adjusted for need (service utilisation), equity (catchment population, remoteness of the health facility) and facility performance (availability of tracer medicines and use of modern family planning methods). PHC providers have been empowered to use the single stream of funding they receive flexibly according to prescribed guidelines and under the oversight of the Health Facility Governance Committee (HFGC). The HFGC has representation from the PHC facility management and the community and is a mechanism to improve accountability to the local community. The funds are accounted for through a single financial management system operated at the provider level. Early assessments have revealed that DHFF has improved accountability of PHC providers, improved availability and quality of PHC services, facility deliveries and availability of medicines.^82^,^85^

The DHFF mechanism was initially used for the HSBF funds, but health insurance reimbursements were subsequently included in the funds flowing through the DHFF. Providers prepare plans and budgets for the consolidated pool of funds, rather than for each stream of funds, allowing them greater flexibility to allocate across the sources to the prioritised health interventions or procurement of medical commodities, under the oversight of the HFGC and the district health management team. Accounting for these funds is through the Facility Financial Accounting and Reporting System which creates visibility on spending and budget management from the facility level to the national level. The DHFF reform was accompanied by increased autonomy at the provider level, with thresholds beyond which approval is needed from the District Medical Officer as an additional level of control.

In effect, Tanzania has implemented two levels of defragmentation. The first level is the consolidation of on-budget support in the HSBF. The second level is at the PHC facility level where different streams of funds are consolidated into one pool for use by the providers. The DHFF reform has been successful in improving financial management systems, increasing availability of health commodities and overall health system performance.^86^,^88^ But more is required to strengthen the HFGC in their roles by continuous training due to high churn of members, and continuous support of facility staff on using the financial systems.^85 89^

## Discussion

The country experiences presented highlight that there are practical steps governments can take to begin to address the negative consequence of PHC financing fragmentation while addressing the root cause remains a longer-term aspiration.

The four country experiences show that focusing on coherence and alignment at the PHC-provider level, through the purchasing function, can be a practical entry point. While we do not have conclusive evidence of the long-term impact of the initiatives presented here, these experiences provide four key lessons that can be relevant across low-income and middle-income countries.

### First: avoid adding new schemes or funding flows that bypass the government systems and worsen fragmentation

Efforts to work around existing systems and schemes have led to duplicative governance and coordination structures, benefit packages, provider payment mechanisms and reporting systems that work against effective PHC delivery. Further, organisational weaknesses at the Ministries of Health have resulted in “silos’’, with numerous vertical programmes with multiple and disconnected agendas. This is worsened when core functions are not well defined or executed, such as data and information system governance, strategic purchasing, strategic planning, monitoring and evaluation. Fragmentation is the natural consequence of the weak governance of the system.^90^

Therefore, a starting place is to avoid introducing new schemes and systems that work in parallel to existing ones, and instead build on and strengthen existing foundations where feasible. Burkina Faso and Tanzania did this by introducing improvements to existing systems to enable the flow of additional funds to PHC providers. Overcoming functional or organisational silos may defragment the health system in a sustainable way. This has implications for external resources and donor-led solutions, how they are channelled and accounted for. There are increasingly loud calls for a different approach to donor funding for health that focuses on consolidated mechanisms that align, strengthen and use domestic, public systems in low-income and middle-income countries.^91 92^ Burkina Faso and Tanzania show how this can be done in practice by channelling external resources through existing government mechanisms.

### Second: actively manage the political economy that worsens fragmentation

The process of defragmentation can be politically fraught given competing interests and power dynamics. If the leadership of the ministry is strong and the path to defragmentation is clearly defined with the participation of all stakeholders, implementation is less difficult and each actor can adapt to the direction set by the MOH. However, this is often not the case and instead, defragmentation is a long-term process requiring patience and ongoing commitment and leadership from the MOH who need to respond to and manage complex political economy dynamics of the interrelationships between the various actors and institutions involved in the policy reform.^93 94^

For example, in Burkina Faso, the World Bank sought to extend the PBF programme which was not aligned with the MOH vision for expanding Gratuité. Between 2019 and 2021, MOH rallied stakeholders and aligned them to their vision and Gratuité objectives for increasing coverage rather than extending the parallel scheme. The technical secretariat of health financing reforms was charged with aligning the vision of expanding Gratuité, within the MOH departments and then with the development partners, an iterative process that took extensive engagement led by local and regional experts over three years.^73^ In Argentina, Plan Nacer/Programa Sumar implementation has been an iterative process entailing engaging provinces in its design phase; gradual expansion in terms of regions, population and services; continual refinement of its design and careful coordination across levels of government. The conditional grant to provinces has been a mechanism to align the provincial level to the central government policies and priorities in Argentina,^79^ but in other settings, it has been viewed as a means to extend central government’s control/influence and undermine devolution.^95^ However, when well designed with clear performance metrics conditional grants can create better alignment in policies and service delivery.^74^,^77^ Its core principle of defining central rules while providing flexibility has enabled the federal MOH to manage provincial diversity effectively. These examples highlight how political sensitivity and understanding can inform technical reform strategies to feasibly address fragmentation.

### Third: strategic purchasing can be an important entry point to defragment systems

Although consolidating pools may be preferable from a purely technical perspective, it is not feasible in many settings to merge schemes. An alternative is to try to reduce the disparities between the schemes through the purchasing function. An example is Thailand’s Universal Coverage Scheme (UCS) which provides similar benefits and uses the same provider payment rates for the informal and formal sector workers that cover the majority of the population.^62^ However, civil servants continue to benefit from better coverage and higher reimbursement rates than the UCS and aligning the benefit package has proven politically challenging.

As shown in the four country experiences, strategic purchasing may provide the opportunity to harmonise entitlements across different population groups and reduce inequitable access to services. This may be achieved for example, by developing a consolidated benefits package that includes services needed by a broad segment of the population, by having an equitable process to expand services, and by extending coverage to groups with fewer entitlements. Using the same provider payment mechanisms across schemes can ensure that at provider level, there are no incentives to treat certain populations better because they have more generous entitlements or higher provider payment rates associated with their scheme.^18^,^21^

As demonstrated in Indonesia with the merger of public health insurance schemes to create JKN, some countries have been able to overcome aspects of fragmentation by merging schemes. This has also been achieved in Ghana and Rwanda where district health insurance schemes and mutuelles were merged to create the National Health Insurance Scheme and Community Based Health Insurance Scheme, respectively.^96 97^ In these examples, purchasing is consolidated in one agency, although other agencies may play complementary roles. Reducing fragmentation in both the pooling and purchasing functions has improved equitable coverage in these countries by ensuring all the beneficiaries have access to the same benefit package, the same choice of provider, with the same provider payment mechanisms. As each provider payment mechanism has pros and cons, blended payment models purposively combine payment methods to maximise beneficial incentives and offset perverse incentives of each payment method, while ensuring service delivery objectives, such as access, are met.^8 98 99^

To overcome the perverse incentives from multiple funding channels, allowing primary care providers to receive funds they can use flexibly may improve PHC service delivery. Primary care providers may either capture all the revenues in a single budget to ease planning and budget execution as in Tanzania, or as in Argentina and Tanzania, expand provider autonomy to use these funds flexibly to facilitate better resource use that matches local priorities.

### Fourth: defragmenting a small part of the system can be an important catalyst to drive more efficient and effective PHC financing

The prospect of merging schemes and creating consolidated risk pools to reduce fragmentation may appear politically and technically daunting. Merging schemes is particularly challenging in devolved settings where different levels of government have responsibility and receive funding for PHC. However, taking an incremental approach, starting with a small pool of funds, and integrating within PFM systems has been one way of catalysing larger system changes. For example, in Argentina’s Programa Sumar conditional transfers linked to results channelled to provinces through existing systems helped to improve equity in resource allocation, align efforts, incentivise better performance and strengthen essential functions of the health system, in a devolved setting. By investing less than 1% of the average annual provincial health budgets, Programa Sumar has made significant contributions to the improvement of both the organisational performance of the health system and health outcomes. Argentina illustrates that in decentralised systems, intergovernmental conditional transfers, if well designed and implemented, may incentivise and require subnational governments to adopt institutional and financing improvements to reduce fragmentation. Whereas through DHFF, Tanzania addressed bottlenecks to resources flowing to frontline providers by creating a single revenue source with supporting systems that balance flexibility with accountability and address inequities and inefficiencies previously observed, when resources were previously ‘stuck’ at the district level. These examples demonstrate that it is possible to work within existing systems rather than creating alternative financing flows thereby harnessing efficiencies, reducing administrative costs while improving equity in resource allocation.

The examples presented here do not address all aspects of fragmentation, for instance, none address the fragmentation caused by vertical health programmes funded by global health initiatives (GHIs) which remains an area for redress and action. The 2023 report on Reimagining the Future of GHIs, along with recent research examining factors the contribute to sustainable transition from external assistance,^92 100^ recommend that this is addressed by GHIs investing in core functions, such as common information systems and drug procurement, and using PFM systems and/or avoiding parallel fund disbursement systems to reduce transaction costs and inefficiencies.

## Conclusion

Fragmentation of PHC financing is one of the most consequential challenges facing countries seeking to improve PHC to achieve UHC, but it remains underexamined. In this paper, we offer some hypotheses on the effects of fragmentation but there is scope for further refinement of the concept of fragmentation of financing, for PHC and more generally, including consideration of when fragmentation is inherently negative, the consequences of fragmentation, and how these can be addressed through policy design illustrated by steps countries are taking to mitigate the negative consequences of fragmentation.

While it is important to address the drivers of low public spending, we recognise this may be slow to change due to macrofiscal and political constraints. We have argued here that there are practical actions countries can take to minimise the consequences of fragmentation—by reducing or avoiding new fragmentation at the level of revenue sources and pooling arrangements and reducing fragmentation at the provider level through purchasing functions. We focus on efforts to improve PHC financing arrangements through improved pooling and harmonised purchasing functions, but we recognise there are other entry points to address fragmentation, such as supply chain, human resource management and information systems.

The long-term impact of some of these country examples remains unclear as they are still in the early stages of implementation, but the lessons may provide a practical way forward for government stakeholders grappling with the consequences of fragmented PHC financing. In addition, political will and leadership are needed to support extensive changes to institutional arrangements and in some cases to create new institutions for consolidation purposes, such as the case of Indonesia. Countries may consider starting by providing flexible funds to PHC providers with a common set of rules for their use, allowing PHC providers to set and resource priorities driven by the needs of their communities, and creating accountability mechanisms for resources they receive and to the community they serve.

The hypotheses and lessons also point to a research and learning agenda to understand both the root causes and the effects of fragmentation to providers, effects on purchasing and the broader health system, and to generate evidence on whether and how defragmentation approaches are effective. In general, the effects of fragmentation have not been extensively studied and there remains a substantial research agenda to understand the enabling factors for defragmentation, the most effective and feasible way to sequence defragmentation reforms, how to navigate the tensions between different funding flows to improve purchasing, and develop a robust evidence base on the levers available to practitioners to reduce fragmentation in their health systems and by so doing, improve equity, efficiency and effectiveness of PHC. In addition, there is a need to test different approaches to reduce fragmentation caused by funding through GHIs and to evaluate these approaches to better prioritise and scale the most effective approaches.

Finally, the lessons provide a clear message to donors and implementing partners who should be aware of the potential harm created by new programmes that worsen fragmentation and to national governments that it is possible to direct external resources through domestic systems to national priorities.

## Data Availability

Data sharing not applicable as no datasets generated and/or analysed for this study. All data relevant to the study are included in the article or uploaded as supplementary information.
